# Single-Cell RNA Sequencing of the Cardiovascular System: New Looks for Old Diseases

**DOI:** 10.3389/fcvm.2019.00173

**Published:** 2019-12-10

**Authors:** Farhan Chaudhry, Jenna Isherwood, Tejeshwar Bawa, Dhruvil Patel, Katherine Gurdziel, David E. Lanfear, Douglas M. Ruden, Phillip D. Levy

**Affiliations:** ^1^Department of Emergency Medicine and Integrative Biosciences Center, Wayne State University, Detroit, MI, United States; ^2^Center for Molecular Medicine and Genetics, Wayne State University, Detroit, MI, United States; ^3^Heart and Vascular Institute, Henry Ford Health System, Detroit, MI, United States; ^4^Department of Obstetrics and Gynecology, Center for Urban Responses to Environmental Stressors, Wayne State University, Detroit, MI, United States

**Keywords:** RNA, cardiology, single cell RNA sequencing, epigenetics, genetics

## Abstract

Cardiovascular disease encompasses a wide range of conditions, resulting in the highest number of deaths worldwide. The underlying pathologies surrounding cardiovascular disease include a vast and complicated network of both cellular and molecular mechanisms. Unique phenotypic alterations in specific cell types, visualized as varying RNA expression-levels (both coding and non-coding), have been identified as crucial factors in the pathology underlying conditions such as heart failure and atherosclerosis. Recent advances in single-cell RNA sequencing (scRNA-seq) have elucidated a new realm of cell subpopulations and transcriptional variations that are associated with normal and pathological physiology in a wide variety of diseases. This breakthrough in the phenotypical understanding of our cells has brought novel insight into cardiovascular basic science. scRNA-seq allows for separation of widely distinct cell subpopulations which were, until recently, simply averaged together with bulk-tissue RNA-seq. scRNA-seq has been used to identify novel cell types in the heart and vasculature that could be implicated in a variety of disease pathologies. Furthermore, scRNA-seq has been able to identify significant heterogeneity of phenotypes within individual cell subtype populations. The ability to characterize single cells based on transcriptional phenotypes allows researchers the ability to map development of cells and identify changes in specific subpopulations due to diseases at a very high throughput. This review looks at recent scRNA-seq studies of various aspects of the cardiovascular system and discusses their potential value to our understanding of the cardiovascular system and pathology.

## Introduction

“*Many possibilities for future applications (of precision medicine) spring to mind: today's blood counts might be replaced by a census of hundreds of distinct types of immune cells …”*

—F. Collins and H. Varmus (2015)

There has been tremendous curiosity to further understand the effect of phenotypic variation in the cardiovascular system and its relation to disease progression. For example, the increased expression of certain long non-coding RNAs and the reprogramming of fetal-heart genes are found to be crucial pathologic processes in the development of heart failure (1–4). The transcriptome is the profile of all RNAs in a sample which essentially communicates gene expression levels thereby mapping the phenotype of the sample. Resulting gene expression profiles derived from RNA sequencing (RNA-seq) allow for the interpretation of molecular differences between cell types or tissues. This allows for a better understanding of gene expression regulation, while also identifying changes in gene expression in differing conditions (e.g., development, response to stimuli, and disease progression).

With the development of advanced RNA-seq techniques to better understand the transcriptome of tissues and individual cells, various groups have been able to produce novel insight in the native heart and associated pathology (1–5). RNA-seq has also led to the discoveries of various non-coding RNAs, including long non-coding RNAs and circular RNAs and their role in regulating cardiomyocyte genes (6–8). However, recent advances in single-cell RNA-seq (scRNA-seq) have, for the first time ever, led to discoveries of phenotypically diverse and complicated networks of cells (aka, the “cellulome”) within cardiac tissue at the single-cell level (9, 10). Usage of scRNA-seq has opened a new field of single-cell level precision to diagnostics and therapeutics to combat cardiovascular disease. This revolution was predicted in the leading quote by Collins and Varmus in their 2015 perspective in the NEJM (11). Therefore, research into the transcriptomic alterations within the diverse “cellulome” of the cardiac network has tremendous translational potential.

scRNA-seq has given us the ability to profile the phenotypes of single cells leading to discoveries of new cellular subpopulations that could contribute to cardiovascular pathogenesis. However, because of the complexity of the data, scRNA-seq findings may be difficult to interpret by clinicians or fit into current knowledge bases (12, 13). This review serves to help both clinicians and basic scientists by (1) briefly explaining what scRNA-seq is and how it can be used in cardiovascular research and (2) highlight recent scRNA-seq studies and their potential implication for present and future understanding of the cardiovascular system and related pathology.

## Single-Cell Transcriptome Data Generation

Compared to the traditional technique of bulk-RNA-seq (Table 1), the main improvement that scRNA-seq achieves is that while bulk-RNA-seq averages gene expression across all cells in a sample, scRNA-seq profiles the transcriptome of each individual cell in the tissue sample. Significantly, this means that scRNA-seq makes high throughput investigations of tissue samples far more specific by visualizing the phenotypes at single-cell resolution.

**Table 1:** Summary of differences between Bulk RNA-seq and scRNA-seq.

	**Goal**	**Protocol**	**Quality control**	**Normalization**	**Analyses**
Bulk RNA-seq	• Measure the average gene expression across the population of cells in a sample • To identify differences between sample conditions	• RNA is extracted from all cells in the sample • Reverse transcription converts RNA to cDNA, facilitates ligation of sequencing adaptors • Amplification	• GC content, presence of adaptors, overrepresented k-mers, duplicated reads • Percentage of reads that map to reference • Reproducibility between replicates	• Batch effect • Between-sample variability: sequencing depth Quantile normalization, spike-ins • Within-sample variability: feature length, library size effects RPKM, FPKM, TPM	• Estimate gene and transcript expression • Differential expression analysis • Alternative splicing
scRNA-seq	• Measure the gene expression of individual cells in a sample • To identify differences between cell types/states	• RNA is extracted from isolated cells, labeled with cell specific identifier • UMIs, spike-ins often included, to account for higher levels of noise • Reverse transcription, amplification similar to bulk protocol	• Reads, number of genes per cell • Percentage of reads that map to spike-ins (if used), percentage of reads that map to mitochondria • QC metrics used in bulk RNA-seq are also examined	• Batch effect and within-sample variability are corrected for similarly to bulk RNA-seq • Between-sample variability methods must additionally account for capture efficiency and dropout sources of noise	• Dimensionality reduction • Identify cellsubpopulations • Differential expression • Pseudotime/trajectory analysis

While there are numerous methods for performing scRNA-seq, all of which follow a general workflow: (1) isolating single cells, (2) capturing RNA, (3) reverse transcribing RNA to cDNA, (4) amplifying the cDNA, (5) library preparation, and (6) sequencing. It is within each of these steps that the variety of scRNA-seq protocols make use of different available technologies and methodologies, which are specifically advantageous to particular experimental designs and goals (13–15). For instance, at the single cell isolation step, a manual isolation technique, like micromanipulation, has advantages for samples of few precious cells, while the more high throughput and cost-effective microfluidics isolation techniques are advantageous for experiments with a large number of cells (13). Another experimental detail to consider when selecting a scRNA-seq protocol is the desired transcript coverage. Protocols that sequence full length transcripts, like Smart-seq2, are more advantageous if the goal of the experiment is to analyze isoform usage or allelic expression. Whereas, 3′ sequencing, as used in protocols like the 10x Genomics Chromium droplet-based method, allows for higher throughput and cost-effectiveness, which makes it more advantageous if the experimental goal is cell subpopulation detection. This is because the plethora of cells that could be sequenced with this method increases resolution for improved cell subpopulation detection thereby making detection of rare cell populations more likely. Additional factors to consider when selecting a scRNA-seq protocol include: the type of RNA of interest in the experiment (polyA + or -), whether spike-ins or unique molecular identifiers (UMIs) can be used, and cost (14, 15).

Despite varying advantages of different scRNA-seq protocols, there are shared challenges. For instance, accounting for library preparation costs and confounding batch effects remain significant barriers for all scRNA-seq protocols. One current improvement to scRNA-seq protocols which addresses these limitations is multiplexing (methods that label cells at the sample-level, allowing for pooling and processing of all the cells in one run). Recently developed techniques include: (1) lipid oligonucleotides which contain sample-level barcodes that incorporate into the plasma membrane of live cells, (2) combinatorial barcoding, where cells are identified through the unique combination of barcodes acquired from multiple rounds of random barcoding, and (3) using naturally variant single nucleotide polymorphism genotypes to distinguish cells' sample of origin, among others (16–22).

Most centrally, these many scRNA-seq methods all function in accomplishing the same aim: to profile which genes are expressed and their level of expression for individual cells, which allows for novel data analyses important for investigating fundamental biological questions.

## Single-Cell Transcriptome Data Analysis

### Data Processing

**Figure 1:**
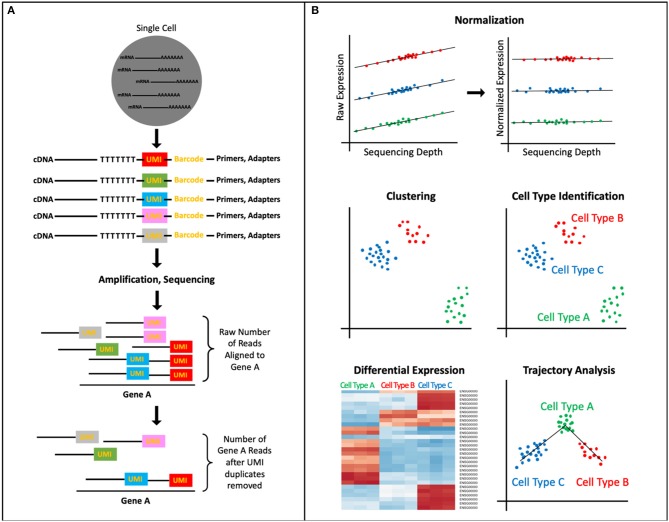
scRNA-seq Data Processing and Analysis. **(A)** UMIs, short DNA sequences tagged to cDNA fragments before amplification, identify unique reads vs. PCR duplicates thereby normalizing the transcript counts. **(B)** A common analysis pipeline for scRNA-seq data includes: normalizing data to account for sources of technical and biological noise (pictured here, sequencing depth), clustering cells to identify novel and known cell types as well as subpopulations, ordering cell types and states into trajectories, and performing differential expression analysis, which can allow for identification of biomarkers, assigning function to cell cluster.

Regardless of the exact protocol followed, all scRNA-seq data present unique interpretational challenges because of the high levels of technical and biological noise (13, 23, 24). RNA capture efficiency, batch effects, transcriptional kinetics, cell cycle stage, and, most significantly, the large amount of amplification owing to the small amount of starting material are a few such sources of noise. Therefore, to ensure that the signal of interest in the data is not masked by unwanted variation, experimental and computational normalization methods have been developed that adjust for these sources of noise in the sequencing data. Typically, multiple methods for removal of noise are used in conjunction because each method accounts for a specific source of bias/noise. For instance, the experimental integration of UMI sequences allows for the detection and removal of amplification duplicates, thereby adjusting read counts across samples (Figure 1A) (25). Another important source of noise is variation in sequencing depth between cells (Figure 1B). Initially, sequencing depth normalization was accomplished through bulk-RNA-seq established methods, like Reads Per Kilobase Million (RPKM), Trimmed Mean of M-values (TMM), and quantile normalization (26–28). Recently, scRNA-seq data specific methods have been developed, like Single-Cell Differential Expression (SCDE) or Model-based Analysis of Single-cell Transcriptomics (MAST), which take into account attributes unique to single-cell expression data, like the high rate of dropout events (genes erroneously reported to have zero expression because of missed RNA capture) (29, 30). These normalization methods are an important preprocessing step to improve the quality of downstream analyses.

The data analysis workflow for scRNA-seq data, as implemented by software packages like Cell Ranger Seurat, and Monocle, includes: (1) elucidating cells' heterogeneity via clustering cells based on gene expression profiles, (2) characterizing cell clusters by assigning cell type or functionality via biomarkers or differential expression analysis, and (3) organizing defined cell types/states into a trajectory (Figure 1B) (31–35). The first step to identify underlying patterns among the transcriptomes of the single cells is to perform dimensionality reduction. Dimensionality reduction tools, like PCA, tSNE, and more recently, UMAP, project the high dimensional scRNA-seq data (the expression levels of thousands of genes per cell, in thousands of cells) into lower dimensional space, thereby collapsing the data and effectively identifying and preserving only the features that contributed to the structure of the original high-dimensional data (14, 36–40). The cells can then be separated into populations based on the similarity of their gene expression profiles through clustering algorithms that employ one of four main methods: k means, hierarchical clustering, density-based clustering, or graph-based clustering (39, 41). The identified clusters can then be visualized via a scatterplot that translates individual cells into data points, where cluster membership is indicated by physical proximity of the points on the plot.

### Data Applications

The processed scRNA-seq data is then suitable for use in analysis applications, such as, characterizing the cell clusters, and trajectory inference. The most straightforward method for characterizing cell clusters is to identify cluster specific expression of cell type biomarker genes. In the case where cells cannot be identified via biomarkers, differential expression analysis, of which the different methods are highly abundant and well-established, presents an alternative method for cell cluster characterization (42). Differential expression analysis identifies sets of genes significantly more highly expressed within clusters compared to all other cells, which can provide clues as to the identity of the cell type/state or the functionality of the cells within the cluster (13, 43). Another analysis of scRNA-seq data is trajectory inference “pseudotime” analysis. This analysis works by ordering cells along a trajectory based on the similarity of their gene expression profiles. Numerous methods have been developed to perform pseudotime analysis, but the most important factor when selecting a method is the type of biological trajectory that is expected (for instance, a linear, bifurcated, or multifurcated cell type differentiation trajectory) (44, 45). Despite the trajectory topology of the method, pseudotime analyses face some limitations that are aimed to be addressed by future method developments, these include: (1) accounting for other processes (like cell cycle stage) that may mask the gene expression patterns of the process of interest and (2) incorporating other types of information (such as location, chromatin state, and post-translational modifications) that contribute to a cell's state in addition to transcriptome (44, 45).

While scRNA-seq data require careful processing to achieve interpretability, the end result of elucidating the cell types/states present in a sample and their relationships to each other (i.e., which genes are differentially expressed between them, and in what order they occur in a dynamic process) has critical implications for deepening our understanding of disease pathologies.

## scRNA-sequencing to Unravel the Hidden Cardiac Cellulome

Many studies prior to the utilization of scRNA-seq have demonstrated the importance of non-traditional cells such as resident fibroblasts, monocytes, and other non-cardiac myocytes within the cardiac network (46, 47). However, it wasn't until scRNA-seq studies of the heart in both murine and human models that the development of a high-resolution map of the non-cardiomyocyte cellulome within the heart was possible (48). scRNA has discovered tremendous heterogeneity within major cell types contributing to the complex map of the cardiac cellulome. The identification of different subpopulations and rare cell subpopulations has a tremendous implication on cardiovascular disease. For example, Skelly et al. discovered hybrid macrophage/fibroblast subpopulations of cells natively residing in the murine heart, which could represent a new class of resident cells that may mediate myocardial fibrosis in response to stress (48). These cells are commonly known as fibrocytes, expressing both fibroblast and macrophage genes, which have been shown to be crucial mediators of chronic inflammatory states (49, 50). It is widely believed that after cardiac injury, infiltrating responders such as neutrophils and macrophages are the mediators of cardiac inflammation and subsequent repair. For example, post-myocardial ischemia and reperfusion, cardiomyocytes undergo significant oxidative stress and neutrophils will be the first responders followed by macrophages, which is crucial to both the inflammation phase and the repair phase of the myocardium. However, with the discovery of new rare resident subpopulations, such as the hybrid macrophage/fibroblast, we can hypothesize that these resident cells may help initiate extracellular matrix remodeling in response to stress prior to inflammatory response.

This subsequent extracellular fibrosis formation following myocardial injury may be reparative or reactive (51). It has been hypothesized that IL-11 is implicated with thrombopoiesis and fibrosis formation (52, 53). The administration of recombinant IL-11 following myocardial infarction was shown to be cardioprotective in a mice model and in a small case-series of patients (54, 55). However, it's exact mechanism of action was unknown. To assess IL-11's role in fibrosis development, Schafer et al. had found that in bulk-RNA analysis of fibrotic human hearts, IL-11 RNA expression was positively correlated with myofibroblast populations. Additional scRNA-seq experiments of fibrosis-susceptible mice confirmed that IL-11 expression by fibroblasts was seen in a subpopulation of fibroblasts that had TGFβ1 activation or had high expression of pro-fibrotic phenotypes. It was shown that IL-11 played an important role in myofibroblast differentiation from fibroblasts, and the activation of these fibroblasts were dependent on IL-11 signaling. This was subsequently confirmed by knocking-out the IL-11 receptor in mice which resulted in attenuation of fibrosis growth in both an angiotensin-II infused model and in trans-aortic constriction model (56). This discovery of IL-11 role in a specific fibroblast-subpopulation during differentiation and activation has supported the notion that targeting IL-11 could be a multi-faceted approach to cardiovascular disease. Positively targeting IL-11 immediately following myocardial injury could promote reparative fibrosis while negatively targeting IL-11, during significant stress (i.e., hypertrophic cardiac disease), could attenuate negative fibrosis.

A recent scRNA-seq experiment on human fetal embryos *in vivo* demonstrated a similar discovery of transcriptome variation in the human cardiac cellulome. The human embryo study identified spatially- and temporally-associated transcriptomic patterns of cardiomyocytes and fibroblasts during development (57). Specifically, expressions in extracellular matrix genes were increased in both cardiomyocytes and fibroblasts, providing strong evidence to the growing theory that both cardiomyocytes and resident fibroblasts contribute to the extracellular formation of the cardiac landscape. scRNA-seq identified unique transcriptomic phenotypes associated with normal human fetal heart development and abnormal fetal heart gene reprogramming seen in heart failure. However, it should be noted that this study found differences in the chronological order of expression of phenotypes in the human heart development as compared to a murine model of development. It was found that the extracellular matrix genes were expressed at higher levels relatively earlier in human cardiac development compared to that seen mice (57). However, the identification of these differences in development and the identification of other phenotypic differences in future scRNA-seq studies could help us identify both strengths and weaknesses of various murine models of cardiovascular disease and cardiac regeneration.

## Phenotypic Heterogeneity of Normal Cardiomyocytes and Pathologic Cardiomyocytes

scRNA studies in the adult heart have elucidated tremendous variation of genetic expression within cardiomyocytes (48). Non-pathologic cardiomyocytes exhibit significant gradients of expression of cardiac markers including actin alpha cardiac muscle 1 and alpha-myosin heavy chain. Significant heterogeneity of these cardiomyocytes at a non-pathologic state is an important finding, considering that in the setting of certain pathological progression there are further heterogenic expressions throughout the myocardium. For example, it has been hypothesized with standard bulk-RNA that there are significant heterogenic expressions in heart failure with the classic fetal reprogramming genes, including *Myh7, Nppa, and Nppb* (58, 59). However, scRNA-seq has been able to discover more heterogenic genetic expression, which was not detected with previous bulk-RNA tissue analyses. This includes discovering significant heterogeneity cardiomyocyte subpopulations expressing long intergenic non-coding RNA (LincRNA), *Gas5 and Sghrt*, in the setting of cardiac hypertrophy in a rat model (6). *Gas5* and *Sghrt* are regulatory LincRNAs that appear to arrest the cell cycle and are found to be key regulators of the cardiac cycle during myocardial stress.

In a pressure overload murine model, during early hypertrophic states, cardiomyocytes analyzed with scRNA-seq expressed mitochondrial biogenesis genes to increase oxidative phosphorylation to compensate for hypertrophy (60). This discovery supports the theory that the increased mitochondrial biogenesis in response to cardiac hypertrophy, leads to an augmented rate of oxidative phosphorylation which could exacerbate oxidative-stress damage in the myocardium. This consequential oxidative stress leads to DNA damage which was shown to activate p53 in the later phases of hypertrophy. Interestingly it was shown in mice that p53-knockout specifically in cardiomyocytes was associated with attenuation of cardiac fibrosis and retained cardiac function after 4 weeks of pressure overload. p53 is commonly known as a tumor suppressing gene that detects DNA damage and prevents cell division in all cells (61). However, it was shown that varying expression of p53 across the myocardium leads to significant cell-cell transcriptional heterogeneity. This transcriptional heterogeneity prevents uniform adaptive hypertrophic programming and activates heart failure-related phenotypes. For example, in response to oxidative stress, the cardiomyocytes had an increased expression of *Nrf2*. Nrf2 is a transcription factor that is activated by oxidative stress and increases expression of antioxidant genes, such as those involved in glutathione biosynthesis (62). These findings were identified in scRNA-seq analyses of cardiomyocytes in humans with dilated cardiomyopathy, showing a similar association of distinct transcriptomes with oxidative stress leading to cardiac dysfunction (60).

The scRNA-seq study by Nomura et al. utilized a cell marker-based pipeline to visualize the heterogenous expression of *Myh7* gene expression after pressure overload due to trans-aortic constriction (TAC) in rats (60). They used single molecule fluorescent *in situ* hybridization (smFISH) with RNAscope to visualize the genetic expression variation within the myocardial tissue. smFISH with RNAscope brings significant clinical value by providing single molecule assessment of RNA biomarkers with less technical difficulty, higher sensitivity and higher specificity when compared to prior *in situ* RNA hybridization techniques (63). RNAscope utilizes a unique probe design that allows for amplified signals while suppressing background noises by targeting a sequence in tandem (64). smFISH was used to bind each individual molecule of *Myh7* mRNA expressed in the cardiac tissue; this approach found that *Myh7* actually inversely correlates with cardiomyocyte size. The utilization of a cell-specific marker in the pipeline helps support a previous study, which claimed that *Myh7* genes were greatly expressed with smaller cardiomyocytes as opposed to larger cardiomyocytes (60). Furthermore, *Myh7* gene expression was found to be greatly expressed in the middle layer of the myocardium after chronic pressure overload. This study helps support the notion that *Myh7* is expressed on a spectrum throughout the myocardium and that some cardiomyocytes will change their phenotype to consume less energy by atrophying and expressing slower contracting *MYH7*, as opposed to cardiomyocytes which showed to have increased mitochondrial activity and oxidative phosphorylation (58, 65). Adding a cell-specific marker such as a smFISH of RNA molecules to the scRNA-seq pipeline brings potentially significant clinical value by giving pathologists new biomarkers for disease analysis and by providing a spatial map of cells-of-interest.

## Implementation of scRNA-seq in Regenerative Cardiology

It has been well-established that the adult myocardium lacks the regenerative capacity as seen in the embryonic myocardium. There has been tremendous effort to study the developing embryonic cardiovascular system to gain insight into the factors associated with cardiac regeneration. Single-cell resolution has allowed us to obtain a far more detailed and more complete picture of cellular transitions during development (66, 67). scRNA-seq has been utilized to better understand the complex genetic regulatory and epigenetic networking of various stages of multipotent stem cell differentiation during heart development. Jia et al. recently identified unknown cardiac subpopulations marked by *Nkx2-5* and *Isl1* expression leading to differences in chromatin site accessibility and progenitor cell differentiation using both scRNA-seq and Single Cell Assay for Transposase-Accessible Chromatin using sequencing (scATAC-seq) in a murine model (66). scATAC-seq has been used to map DNA regulatory variations of accessible genomes within individual cells. They identified that the posterior *Hox* gene was temporarily expressed in Is1+ cells in the early stages of heart development in addition to the anterior *Hox* gene. This varying expression of the posterior *Hox* gene is believed to contribute to developmental heart patterns. Furthermore, Nkx2-5+ cells were found primarily in progenitor cells destined to become a part of the cardiac endothelium and smooth muscle cells that are characteristically found in immature cardiomyocytes. Both the *Nkx2-5* and the *Isl1* expressing progenitor cells have tremendous phenotypic heterogeneity within their respective populations.

Recently, Xiong et al. expanded on the phenotypic heterogeneity of *Nkx2-5 and Isl1* in embryonic heart development by using a Cre-LoxP system to longitudinally track their expression in conjunction with scRNA-seq (67). They were able to provide, for the first time ever, a multi-dimensional map of first and second heart field trajectory during development. They showed that the first heart field progeny commits directly to cardiomyocyte differentiation while second heart field progeny undergo a distinct stepwise transition where each subsequent progeny becomes increasingly more restricted in lineage differentiation. The discovery of these phenotypically diverse subpopulations, and their unique path to development could explain why these progenitor cells are able to make developmental decisions contributing to heart development. This may provide crucial information for improving implantation of stem cells for cardiac and vascular regeneration.

## scRNA Implications on Stem Cell Therapy

The myocardial cellulome is an intricate and complex network of functionally intercalated cardiomyocytes and supporting cells in the extracellular matrix. Therefore, transplantation of stem cell-derived therapies to replace damaged cardiac tissue has been severely limited in clinical implementation (68). The implanted human pluripotent stem cells often have difficulty assimilating to the host cardiac network, which can result in arrhythmias and diminished cardiac function (69, 70). It is thus important to better characterize how different stem cell lines differentiate at a single-cell level, as stem cells are highly plastic and can easily differentiate inappropriately to their surrounding environment following transplantation. A recent study utilized scRNA-seq to characterize the multiple lineages derived from multipotent-cardiac progenitor cells to validate if their novel protocol could reproducibly differentiate different stem cell lines (71). They first identified that laminin-221, a basement membrane structural protein isotype, was found abundantly in human cardiac muscle. To confirm that laminin-221 was the most relevant laminin in cardiac differentiation, they quantified the gene expression of various laminin genes at different time points of embryonic development in a fetal mouse heart model. They found that laminin-221 gene expression was the highest during development. They then created a recombinant version of this laminin isotype and seeded two different human embryonic stem cell lines (H1 and HS1001) in a culture containing this isotype for differentiation. It was shown that a laminin-221 coating could reproducibly differentiate these different cell lines at different time points. They used scRNA-seq and bulk RNA-seq to validate the transcriptional phenotypes at the different time points to validate this reproducibility. The study injected two different stem cell lines (H1 and HS1001) in the mice myocardium following myocardial ischemia prior to reperfusion and showed improved cardiac function and formation of new human muscle fiber bundles *in vivo*. The usage of scRNA-seq could be a new and more specific benchmark to validate the reproducibility of different stem cell differentiation methods prior to transplantation.

Induced pluripotent stem cells (iPSC) allows for the development of *in vitro* cell lines which can closely mimic the genetic basis for cardiovascular disease (72). Studies have utilized iPSCs to better understand structural heart, arrhythmic, and vascular disorders (73). However, as seen with embryonic pluripotent stem cell-derived cardiomyocytes, iPSC-derived cardiomyocytes also do not match the mature *in vivo* counterpart (74, 75). Therefore, defining the signaling pathway of iPSC-to-cardiomyocyte transition is critical to improve the clinical application of iPSC. Churko et al. utilized scRNA-seq on a heterogeneous cardiomyocyte population derived from iPSCs at different time points of differentiation (76). They identified that after day 30 of differentiation, there were multiple unique subpopulations enriched with several transcriptional factors. These transcriptional factors had a temporal expression pattern during differentiation that correlated with unique subpopulations. For example, in conjunction with chromatin immunoprecipitation sequencing (ChIP-seq) analysis, they provided evidence that *NR2F2* vs. *HEY2* have an important transcriptional regulatory role in promoting atrial functional cell states vs. ventricular cell states, respectively. They confirmed this finding by ablating Nr2f2 in mice fetal myocardium which resulted in ventricular transformation of atrial tissue. Identifying the temporal transition of iPSCs to cardiac tissue and its associated chromatin regulatory network provides potentially valuable information to optimize iPSC-differentiation protocols. Although this study only characterized this differentiation process *in vitro*, which can result in significant differences in genetic expressions and subcellular trajectory as compared to that seen in an *in vivo* environment, targeting this transcriptional regulatory network could promote homozygosity of iPSC differentiation (77). For example, it has been shown that increasing retinoic acid to promote *NRF2* expression improves atrial-specific differentiation from iPSCs as compared to ventricular differentiation (78).

## New Insight in Coronary Vasculature Development With scRNA-seq

Single-cell resolution mapping has been applied to coronary vasculature development as well. It is known that the heart contains a vastly heterogenous microvascular network, and knowledge surrounding this network and its implication in disease remains limited (79). This heterogeneity contributes to a mosaicism of oxygen delivery to various parts of the myocardium and larger arteries (80). This heterogeneity could contribute to the variability of outcomes seen post-myocardial infarction in our patient population (81, 82). Additionally, areas of myocardial damage after infarction are known to undergo angiogenesis in response to chronic hypoxic stress associated with subsequent fibrosis formation (82, 83). Therefore, the formation of the coronary vasculature and its associated microvasculature could be of significant clinical importance. During embryologic development, it has been suggested that premature veins could be reprogrammed to become coronary arteries (84). However, the transcriptional process of vein-to-artery switching had not been characterized. Su et al. utilized scRNA-seq techniques to identify the transcriptional phenotypes of premature vein cells within the developing murine heart that would eventually undergo a vein-to-artery switch independent of coronary blood flow (85). Previously, it was believed that the development of the coronary vasculature was only initiated in connection with aortic trunk, which would then provide embryonic blood flow (86, 87). scRNA-seq was able to illustrate that premature vein precursors would switch gradually from the venous gene phenotype to an arterial phenotype independent of coronary blood flow (85). New knowledge obtained from this experiment could help elucidate cardiac angiogenesis associated not only in embryological heart development, but also in pathology.

As sudden death from myocardial infarction (MI) has decreased and more people are surviving MIs, there is a growing clinical need to attenuate the damage following MI to improve outcomes and prevent heart failure. Various strategies have been employed to reduce infarct size and preserve or even improve cardiac contractility (88–90). Neovascularization of coronary arteries at the infarct border following MI has been shown to improve myocardium outcomes (91, 92). It has been demonstrated that resident endothelial cells contribute to neovascularization following MI via clonal expansion (93). However, migration of bone marrow cells and the endothelial-to-mesenchymal transition of endothelial cells have also been implicated as promoters of neovascularization following cardiac injury (94). Li et al. preformed a multispectral lineage-tracing mouse model on endothelial cells along the infarct border following MI (95). They showed with scRNA-seq that there are 10 transcriptionally distinct states for endothelial cells during clonal expansion in neovasculogenesis. Furthermore, they identified that bone marrow-derived and endothelial-mesenchymal transitioning of endothelial cells were not implicated in neovascularization at the infarct border. This study provides strong evidence for the clonal expansion of endothelial cells and weakens the possibility that bone marrow cells and endothelial-mesenchymal transitioning play a significant role in neovasculogenesis. Their finding is supported by the fact that clinical trials using autologous bone marrow transplantation to promote recovery following MI have failed (96). Additionally, Li et al. were able to give high resolution information on the hierarchical transcriptional states of endothelial clonal expansion. This information could lead to therapeutic targets to promote neovasculogenesis following MI and thus potentially improve prognosis (95).

## Phenotypic Heterogeneity Associated With Atherosclerosis

Atherosclerosis has long been identified as an inflammatory response within the subendothelial space of arteries. However, atherosclerotic plaques are extremely heterogenous with different types of plaque formation, different growth rates, and differences in susceptibility to rupture (97). Furthermore, this heterogeneity among plaque formation is compounded by a phenotypically diverse environment of cell responders within the plaque. The plaque, in and of itself, involves an extremely complicated system of cell-cell and cell-particle interactions which leads to progression and sometimes rupture (98). scRNA-seq has the potential to better deconvolute the complexity of the cellular phenotypes seen within atherosclerotic plaques, including mediating inflammatory cells (99). This approach has great potential to increase our understanding of atherosclerotic plaque formation. Recent scRNA-seq studies have better characterized leukocyte subpopulations in murine models of atherosclerosis (100, 101). It is known that monocytes differentiate into different types of macrophages such as M1 or M2 macrophages. M1 macrophages are considered generally pro-inflammatory, while M2 macrophages are considered anti-inflammatory (102). The transition of monocyte to these different macrophages is associated with plaque formation or regression (103, 104). Recent scRNA-seq findings in mice illustrate that (1) although M1 and M2 macrophage cells are the predominant immune cell in atherosclerotic plaques, there are many different immune cells that overlap expressions, (2) there is a spectrum of types of activation for macrophages, and (3) there are new subtypes of macrophages which we can identify in association with atherosclerotic plaques (100, 101). It was shown that a new species of macrophage clusters expressing TREM2 is highly expressed in atherosclerotic plaques (100). These special macrophages are believed to have specialized lipid metabolism and catabolism functions. Decreased plaque-expression of TREM2 has been associated with plaque instability in human carotid artery samples (104). Interestingly, this may be related to the fact that these macrophages also had the phenotypic expression similar to that seen in osteoclasts, implicating TREM2 macrophages in complicated atherosclerotic plaques with calcification (105, 106). It has been shown that osteoclast-like cells in plaques have a reduced capacity to degrade mineral deposition and prevent calcification compared to that seen from skeletal osteoclasts (106, 107). Although, large calcifications are associated with stabilization of plaques, early micro-calcifications have been implicated in plaque-instability and potential rupture (108, 109). Therefore, TREM2 expression may be a key factor in plaque pathology and needs to be further studied in the context of different stages of calcification.

In addition, phenotypic mapping by scRNA-seq was able to further elucidate the transcriptional overlap between macrophages and dendritic cellular phenotypes, which has been shown to contribute to early atherosclerotic formation (100, 101, 110). The significance of the monocyte- dendritic cell phenotype overlap in plaque formation is unknown. However, it could be hypothesized that overall increase in expression of dendritic-like phenotypes in macrophage populations helps promote the long-term adaptive immune inflammation seen in atherosclerotic plaques (104, 111).

Among the various phenotypic expressions of different macrophage subtypes, it was shown in one scRNA-seq study that non-foamy macrophages expressed more of an inflammatory phenotype when compared to foamy macrophages (110). Non-foamy macrophages expressed higher levels of cytokines (e.g., IL-1b), which is the target of interest for canakinumab in reducing cardiovascular mortality in the ongoing CANTOS trial (NCT01327846) (112). This discovery introduces new questions considering that it was conventionally believed that foamy macrophages were the drivers of lesion inflammation (113). This scRNA-seq study helps confirm Span et al. which challenged the notion that foamy macrophages contributed to inflammation of atherosclerosis by showing that foamy macrophages had a deactivated inflammatory response instead of an activated pro-inflammatory response (114). However, more scRNA studies must look further into dividing foamy from non-foamy macrophages in human plaque samples since these previous studies used knock-out mouse model, which is pathologically different to human atherosclerotic plaque formation (115).

scRNA-seq has also been used to characterize other non-inflammatory cells involved in atherosclerosis (116, 117). Vascular smooth muscle cells (VSMCs) have been shown to be significantly heterogeneous in blood vessels with direct implications to atherosclerotic progression (118). Dobnikar et al. utilized scRNA-seq of adult mouse aortas and identified a rare subpopulation of Myh11-lineage VSMCs that express the multipotent progenitor marker Sca1 (116). These rare VSMC cells were shown to decrease contractile-related gene expression yet increase gene expression related to response to inflammation and growth factors. Dobnikar et al. subsequently showed that Sca1+ VSMCs had a phenotypically similar transcriptional profile in both healthy vessels and in atherosclerotic vessels. Sca1 was not found in VSMC-derived plaque cells that displayed a contractile signature and was also absent in VSMC-derived plaque cells that already gained other cell-types signatures such as those from chondrocytes. Therefore, the authors propose that *Sca1* expression could indicate an intermediate step for VSMC plasticity in plaque development. Although Sca1+ cells have been identified in bulk-blood vessel tissue before, scRNA-seq allowed for the identification of Sca1+ in a rare subpopulation of VSMCs, therefore providing higher-resolution insight into atherogensis (119).

Based on the findings from Dobnikar et al. upregulation of *Sca1* in specific VSMCs could promote a process called phenotypic modulation (116). Phenotypic modulation in the setting of vascular disease, is when VSMCs differentiate, proliferate, and migrate in response to pro-atherogenic stimulation (120). It has been hypothesized that mediating phenotypic modulation could attenuate or even prevent atherogenesis leading to vascular disease (121). It has been proposed that phenotypic modulation could be crucial in coronary artery disease by either maintaining plaque stability or by destabilizing the plaque leading to rupture (120, 122). However, identifying the exact transcriptional changes involved in phenotypic modulation have been difficult *in vivo* because there is a low expression of canonical VSMC markers and there are other cell types that express common VSMC markers (123–125). Recently, scRNA-seq of mice aortas and dissociated human coronary arteries have identified the trajectory of contractile VSMCs toward a fibroblast-like cell called a fibromyocyte (117). This phenotypic trajectory is the opposite of that seen with myofibroblast trajectory where a fibroblast acquires the contractile properties of a muscle cell (126). VSMCs which undergo phenotypic modulation may genetically shift their expression to fibroblast-like cells under the positive-regulation of *Tcf21*. Genome-wide association studies (GWAS) demonstrated that genetic variants of *Tcf21* is causally associated with coronary artery disease, however, scRNA-seq may have elucidated a significant role for *Tcf21* in phenotypic modulation (117, 127). This was the first study showing a GWAS-identified gene mediating SMC phenotypic modulation *in vivo* in the setting of coronary artery disease. The authors concluded that since*Tcf21* was associated with reduced risk of coronary artery disease, and that *Tcf21* expression increased phenotypic modulation, phenotypic modulation is most likely associated with reduced coronary artery disease. It can be hypothesized that *Tcf21* promotes VSMC to fibromyocyte transition thus augmenting fibrosis formation within the atherosclerotic lesion and the fibrous cap. This theoretically would stabilize the plaque from rupturing leading to an acute cardiovascular event. Although further studies need to be performed to investigateTcf21's atheroprotective potential, promoting Tcf21 activity could be an interesting approach to reduce the incidence of plaque rupture.

## Limitations and Future Directions

We should note that many researchers have identified potential limitations to scRNA-seq data. It has been pointed out that the samples utilized for scRNA-seq do not capture the longitudinal expression of genes within the cardiac cellulome and that stochastic changes overtime could cause significant transcriptional variation (128, 129). The issue becomes compounded by the fact that scRNA seq of the heart is only able to pick up 5–20% of the entire transcriptome per cell (128). Furthermore, extracting single cells from cardiac tissue is poor due to suboptimal dissociation methods currently available leading to relatively lower quantities of cells available for scRNA-seq (128). This greatly reduces the power of scRNA-seq for cardiac tissue. These variations and relatively low coverage in heart samples could reduce the accuracy and sensitivity of determining the underlying transcriptional phenotype associated with cardiac disease, especially of those with weakly expressed genes (129, 130). A potential way to avoid this issue is to analyze scRNA data longitudinally in order to identify stochastic noise that may produce different results from single-time point studies. However, since scRNA-seq is highly expensive and technical difficulty, widespread usage has been limited. It currently costs thousands of dollars and takes a team of experienced scientists to run and analyze a single sample from a single patient; therefore, doing larger-scale single-cell experiments are unfeasible at the current time (14). Improvements in RNA capture efficiency, cell dissociation, and sequencing depth will drive scRNA-seq to mainstream usage (14). Currently, scRNA-seq with combinatorial-indexing (sci-scRNA-seq) utilizes split-pool barcoding of cells or nuclei (131). This method has been used to analyze multiple organs at once from mice embryos (132). Sci-scRNA-seq allows for researchers to characterize the phenotypic variation at a multi-organ system level thus giving potentially novel insight into diseases with pleiotropic implications. Although sci-scRNA-seq is still in its infancy, it has major potential for improving coverage depth of tissue analysis and for reducing cost.

Though, as was seen with the development of next generation sequencing, the current peak interest in single-cell technologies will result in rapid advancements in the field leading to more widespread use. Therefore, current cardiovascular scientists and cardiologists should become aware of its potential impact to the future of cardiovascular medicine (Table 2). Through further application of this technology, the future holds many exciting opportunities for adding novel insights into the cardiovascular field. Single-cell transcriptome profiling would be instrumental in characterizing the transition trajectory of cells through subtypes and states during disease development and progression. Future scRNA-seq studies could eventually lead to identifying disease-promoting phenotypes for more precise therapeutic targeting and diagnostic markers. This could be particularly poignant, considering the possibilities: for monocyte subtypes, which have been shown to both promote and attenuate atherosclerosis; for fibroblast-like cell subtypes, which have been implicated in worsening of cardiac fibrosis and heart failure. scRNA-seq would be invaluable for investigating subpopulation variation and cell-differentiation in cardiovascular disease and fetal heart development. scRNA-seq has and will continue to deepen our understanding of various cardiovascular diseases which will undoubtedly improve the precision of our patient care.

**Table 2:** Summary of main studies.

**Study**	**Tissue**	**Number of cells analyzed after quality control filtering (rounded to nearest whole number)**	**Platform**	**scRNA-seq significance**
Skelly et al. (48)	Mouse heart	10,519	10x genomics chromium	Characterized the immense heterogeneity of the non-myocyte cardiac cellulome
Schafer et al. (56)	Mouse heart (fibroblasts)	1,263	10x genomics chromium	Identification of an upregulation of Il-11 in cardiac fibrosis-prone PLN^R9C/+^ mice
Cui et al. (57)	Human fetal heart	3,842	STRT-seq	Characterized human fetal cardiac development
Nomura et al. (60)	Mouse heart and human heart	396	Smart-seq2	Identifying the heterogeneity of cardiomyocyte gene expression in response to pressure overload
Jia et al. (66)	Mouse fetal heart	421 (Fludigm C1) 663 (WaferGen iCell 8)	Fludigm C1 and WaferGen iCell8	Reconstruction of developmental trajectories in cardiogenesis and their association with different chromatin states
Xiong et al. (67)	Mouse fetal heart	616 average for each group	Smart-seq2	Creation of a multi-dimensional map of the intercommunication between first and second heart fields during development
Yap et al. (71)	HS1001 and H1 cell lines	695 average for each group	10x genomics chromium	scRNA-seq was used to assess the reproducibility of a stem-cell differentiation method
Churko et al. (76)	Human iPSCs	10,376	10x chromium	Identification of the transcriptional regulatory network in cardiomyocyte subpopulation differentiation from iPSC
Su et al. (85)	Mouse fetal coronary vessels	334 average for each group	Smart-seq2	Identification of novel developmental trajectories for embryonic coronary arteries
Li et al. (95)	Mouse heart	3,575 average for each group	10x genomics chromium	Identification of a subpopulation of resident endothelial progenitor cells that mediate neovasculogenesis following myocardial infarction
Cochain et al. (100)	Mouse aorta	854	10x genomics GemCode	Characterized the transcriptional heterogeneity of aortic macrophages and monocyte-derived dendritic cells in a mouse atherosclerosis model
Lin et al. (101)	Mouse aorta	2,678 average for each group	10x genomics chromium	Profiling the spectrum of macrophage activation states
Kim et al. (110)	Mouse aorta	10,000 average for each group	10x genomics chromium	Identified that nonfoamy macrophages had more inflammatory characteristics than that seen with foamy macropahges
Dobnikar et al. (116)	Mouse aorta	143 (Fludigm C1) 150 (Smart-seq2) About 2800 (10x Genetics Chromium)	Fludigm C1, Smart-seq2, and 10x Genetics Chromium	Detection of a rare population of potentially atherogenic-prone Sca1+ VSMC cells in healthy mice aortas
Wirka et al. (117)	Mouse aorta and human coronary arteries	About 3,500 cells	10x genomics chromium	Identification of Tcf21 as a pro-phenotypic modulator which was associated with protection from coronary artery disease.

## Author Contributions

FC and JI contributed equally to the creation of this review. FC, JI, TB, DP, KG, DL, DR, and PL were all contributors to various parts of the review and helped in the writing of the review.

### Conflict of Interest

The authors declare that the research was conducted in the absence of any commercial or financial relationships that could be construed as a potential conflict of interest.
